# The Draft Genome of *Cryptocaryon irritans* Provides Preliminary Insights on the Phylogeny of Ciliates

**DOI:** 10.3389/fgene.2021.808366

**Published:** 2022-01-12

**Authors:** Yulin Bai, Zhixiong Zhou, Ji Zhao, Qiaozhen Ke, Fei Pu, Linni Wu, Weiqiang Zheng, Hongshu Chi, Hui Gong, Tao Zhou, Peng Xu

**Affiliations:** ^1^ State Key Laboratory of Large Yellow Croaker Breeding, Ningde Fufa Fisheries Company Limited, Ningde, China; ^2^ State Key Laboratory of Marine Environmental Science, College of Ocean and Earth Sciences, Xiamen University, Xiamen, China; ^3^ Biotechnology Institute, Fujian Academy of Agricultural Sciences, Fuzhou, China; ^4^ Fujian Key Laboratory of Genetics and Breeding of Marine Organisms, College of Ocean and Earth Sciences, Xiamen University, Xiamen, China

**Keywords:** cryptocaryon irritans, oxford nanopore technologies (ONT), illumina, draft genome, phylogenomic analysis

## Introduction

Ciliates are one of the most diverse, highly differentiated and ancient groups of microbial eukaryotes (Coyne et al., 2011). The characteristics of nuclear dimorphism (one large macronucleus and one small micronucleus) make ciliates considered to be an excellent model organism in the genetic investigation (Juranek and Lipps, 2007). Some major diseases in marine fish are caused by ciliates, which can cause skin damage, bacterial infection, and even death of the host (Wei et al., 2018; Zhao et al., 2021b). Genomic research on these pathogens is crucial to finding new treatments. It is particularly attractive to identify genes involved in unique metabolic pathways, pathogenicity, and parasite evasion of immune defense mechanisms (Wei et al., 2018).

The ciliated protozoan *Cryptocaryon irritans* is an obligate ectoparasite of marine fish, and its phylogenetic classification has always been controversial (Wright and Colorni, 2002). Due to the high affinity of the morphological characteristics, life cycle and pathogenic mechanism with *I. multifiliis*, *C. irritans* was originally included in the class Oligohymenophorea and also named “Marine Ich” (Corliss, 1979). After comparing the partial 18s rRNA sequence, Wright and Colorni (2002) indicated that *C. irritans* is taxonomically distinct from *I. multifiliis* and justify *C. irritans*' inclusion into the order Prorodontida within the Class Prostomatea (Wright and Colorni, 2002). Parasites usually have relatively complex phylogenetic relationships, and the lack of research on genetic tools such as genomes is the main reason that hinders the development of related biological problems (Ajioka et al., 1998).

Cryptocaryonosis, caused by *C. irritans*, has an extremely wide host range and is responsible for large-scale death of natural populations (Bai et al., 2020), which is one of the most important parasitological problems in marine aquaculture and poses a significant threat to the aquaculture industry (Zhao et al., 2021a). Several strategies for cryptocaryonosis control have been reported, such as antibiotics, vaccines and metal ions, however, they have shown only partial efficacy under field conditions (Yin et al., 2016; Yogeswaran et al., 2010). In addition, the lack of genomic data has hampered the use of molecular tools in developing control strategies for cryptocaryonosis (Kumar et al., 2020). Transcriptome projects have provided partial sequences of many protein-coding genes (Chi et al., 2020; Yogeswaran et al., 2010), but it is not sufficient to support the physiological metabolism and pathogenic mechanism of *C. irritans*. Therefore, full genome sequence is necessary to perform such analyses.

The parasitic lifestyle, bacterial contamination and other environmental factors always result in a complex sample background, which contributes to contamination in DNA preps (Pan et al., 2019). The limitations on the ability to extract quality DNA with sufficient yields for high-throughput library construction, especially considering the loss of DNA associated extraction and purification step, has likely been the greatest barrier to non-model ciliate genome research (Miller et al., 1999). With the popularization of genome sequencing technology, the genome sequences of the known hosts and closely related co-living species have been sequenced and thoroughly annotated (Chen et al., 2019b; Mao et al., 2013), we have been able to assemble and explore a substantial portion of the genome of *C. irritans*.

In this report, we provided a draft genome of *C. irritans* using Oxford Nanopore Technologies (ONT). We assembled the genome sequences into 2,384 contigs with a total length of 45.61  Mb and a contig N50 length of 21.24  Kb. Furthermore, we identified 4.02  Mb (8.81% of the assembly) of repeat content, 8,729 protein-coding genes and 490 ncRNAs. The first *C. irritans* genome is essential to support the basic genetics and molecular mechanisms studies, and will also provide an important resource for the analysis of host-parasite interaction mechanism.

## Materials and Methods

### Sample Collection and DNA Extraction

**FIGURE 1:**
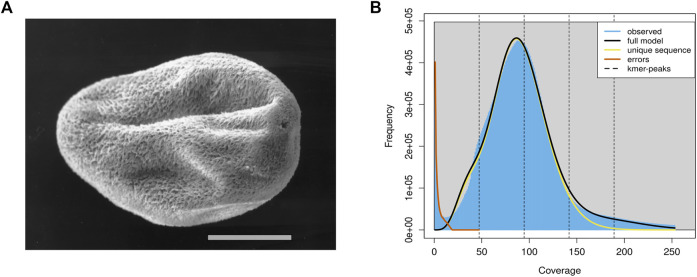
The schematic diagram and genomics feature of the *C. irritans*. **(A)** Pro-tomont of the *C. irritans*. Scale bar 100 μm [adopted from (Bresciani and Buchmann (2001)]. **(B)** A K-mer analysis of the genome sequencing reads for the *C. irritans* using GenomeScope.

*C. irritans* was isolated from an infected *L. crocea* and propagated by passage on juvenile *L. crocea*. Tomonts (Figure 1A) were collected from the bottom of the experimental tank and incubated overnight at room temperature (25–28°C). Theronts from a single beaker were then used to infect a large yellow croaker and offspring from the infection were subsequently maintained by serial passage on fish. Then, all the fish were euthanized by using tricaine methanesulfonate (MS-222; Sigma, St. Louis, MO, United States), and the tomonts were collected, snap frozen in the liquid nitrogen and stored at −80°C.

The harvested cells were washed by low-speed centrifugation through a 0.25 M sucrose solution, then homogenized using a pestle for a 1.5 ml microcentrifuge tube in 0.25 ml of lysis buffer (10 mM Tris; 0.5 M EDTA; 1% SDS; pH 9.5). Proteinase K (0.2 mg/ml) and RNase (40 μg/ml) were then added to digestion. After phenol/chloroform extraction, DNA was dialyzed against Tris–ethylenediaminetetraacetic acid (Tris-EDTA) followed by ethanol precipitation. Nucleic acid concentrations were quantified using a Qubit fluorometer (Thermo Fisher Scientific, Waltham, MA), and then checked by 1.5% agarose gel electrophoresis stained for integrity.

### Library Construction and Sequencing

Paired-end sequencing library with a 350  bp insert size was constructed according to the manufacturer’s instructions. The library was then sequenced with a paired-end sequencing strategy using the Illumina HiSeq 2,500 platform, and the read length was 2 × 150  bp.

Nanopore sequencing library construction and sequencing were conducted according to the manufacturer’s protocol with the Oxford Nanopore MinION platform at Novogene (Tianjin).

### Data Filtering and Genome Survey

Before assembly, the Nanopore data was filtered using NanoFilt (v. 2.8.0) and NanoPlot (v. 1.33.0) software, and the reads with length less than 2000bp or mean quality score less than 10 were removed. For the Illumina data, adapter sequences and low-quality reads were filtered using fastp (v. 0.23.1) software. The remaining reads were used for further assembly and estimation of genome size using the K-mer analysis of the short reads.

In order to obtain information such as genome size, heterozygosity, and repeatability, SOAPec (v. 2.01) and GenomeScope (v. 2.0) softwares were used to analyze the K-mer frequencies in the sequencing reads to efficiently estimate the major genome characteristics.

### DNA Contamination Filtration and *de novo* Assembly of the *C. irritans* Genome

To improve continuity and accuracy of protozoan assembly, we filtered the data in multiple ways and performed hybrid assembly (Florencia et al., 2019). First, nanopore data were mapped to the genome sequence including common marine bacteria and *L. crocea* to filter out the DNA contamination of symbiotic bacteria and the host. In order to correct the over-filtered data, Nanofilt (v. 2.8.0) and Minimap (v. 2.17) sorftwares were used to retain the reads containing GC ratios less than 30% or accurately mapped to the genomes of closely homologous species were retained (Coyne et al., 2011). Then, the filtered nanopore data were assembled into contigs by Flye (v. 2.9) with the parameter “--nano-raw”. After that, the preliminarily assembled contigs were polished by NextPolish (v. 1.4.0) and Racon (v. 1.4.3) software to correct base errors caused by Nanopore sequencing. Finally, we employed Purge_Dups (v. 1.2.5) to resolve redundancy in the assembly.

The Benchmarking Universal Single-Copy Orthologues (BUSCO) software (v. 5.0.0) was used to evaluate the completeness of assembly with the alveolata_odb10 database.

### Annotation of Genomic Repeats

A combination of *de novo* and homology-based predictions were used to identify repeat sequences in the *C. irritans* genome. Firstly, RepeatModeler (v. 2.0.1) and LTR_Finder (v. 1.07) were used to detect repeat sequences in the *C. irritan* genome. Combined with Repbase (20181026), a repeat sequence library was constructed. Then, we used RepeatMasker (v 4.1.0; setting “-nolow -norna -no_is” parameters) to detect and classify repeats based on this library. TEclass (v. 2.1.3) was used to further annotate unclassified repeats. TRF (v. 4.09) was used to identify tandem repeats. Before gene prediction, all regions of repetitive elements were soft-masked with RepeatMasker (v. 4.1.1).

### Protein-Coding Gene Finding and Function Annotation

Both homology-based, *de novo* and RNA-seq strategies were used for gene structure prediction of the *C. irritans* genome. As in the case of other ciliates, *C. irritans* translates the TAA and TAG as glutamine instead of termination codons (Hatanaka et al., 2007), so we adjusted some gene structure annotation software parameters. For homology-based annotation, the protein sequences of three closely homologous species (*T. thermophila, I. multifiliis* and *P. tetraurelia*) were downloaded from NCBI and provided to the exonerate software (v. 2.4.0; setting “--geneticcode 6” parameter) to obtain an accurate gene structure for each locus. To train gene finding algorithms, a set of complete gene structures was modeled manually using the Illumina EST alignments to predict genes of *C. irritans* genome. Then, this set was used to train the *de novo* prediction software Augustus (v. 3.4.0) to predict the gene structure based on the repeat-masked genome. The latest RNA-seq data of *C. irritans* were downloaded from NCBI (SRA accession number: PRJNA600221) and mapped to *C. irritans* genome using PASA (v. 2.4.1; setting “--GENETIC_CODE Tetrahymena” parameter) and Stringtie (v. 2.1.4). The transdecoder software (v. 5.5.0; setting “-G Tetrahymena” parameter) was used to predict gene structure based on ESTs evidence. Finally, evidence from the gene finders, protein homology searches and ESTs were used to refine gene models using EvidenceModeler (v 1.1.1; with the “--stop_codons TGA” parameter). After extensive manual annotation of selected genes, a comprehensive and non-redundant gene set was generated.

For gene function annotation, we used Diamond (v. 2.0.6) to align the candidate sequences to the Swiss-Prot, TremBL and NR protein databases with e-values < 1E-5. InterProScan (v. 5.52–86.0) software was used for GO annotation and protein family annotation. KO terms for each gene are assigned by an online website (KAAS, https://www.genome.jp/tools/kaas/).

The programs tRNAScan (v. 2.0) and RNAmmer (v. 1.2) were used to predict tRNA and rRNA, respectively, and other ncRNAs were identified by searching against the Rfam database (http://eggnogdb.embl.de/).

### Gene Components Distribution and Phylogenetic Analysis of *C. irritans*

To reveal the phylogenetic relationships and gene components distribution patterns among *C. irritans* and other species, we download the protein sequence of *P. falciparum* (outgroup), *P. persalinus*, and *S. lemnae* in addition to *T. thermophila, I. multifiliis and P. tetraurelia*. These genomes were annotated using the same pipeline, and protein sequences shorter than 50 amino acids were removed. Then, in-house scripts are used to count and plot the gene components. OrthoMCL (v. 6.6) and Diamond (v. 2.0.6) software were used to construct gene families from protein sequences of all species, and single-copy genes are identified based on the gene families. Single-copy ortholog proteins were aligned by MUSCLE (v. 3.8.31). Finally, we combined all the translated coding DNA sequences of each species into a continuous ultra-long sequence and used RAxML (version 8.2.12) software to generate a phylogenetic tree.

## Results and Discussions

In total, approximately 8.84 Gb raw illumina data and 16.45 Gb Nanopore reads were generated. After quality filtering, 8.82 Gb clean Illumina data and 12.5 Gb of trimmed Nanopore reads with a mean read length of 8.5 Kb were obtained. For the genome survey analysis, the number of 17-mer was 44,364,461,437, K_depth was estimated as 92.6, GC content and heterozygosity were about 26.86 and 0.5%, respectively. And the corrected genome size is estimated to be 45.67 Mb (Supplementary Table S1), which is similar to the *I. multifiliis* genome size (Coyne et al., 2011). A common single-peak pattern was observed in the K-mer frequency distribution analysis, indicating that the genome may have a low level of heterozygosity and repetitive regions (Figure 1B).

Affected by contamination and heterozygous region, the initial assembly result is larger than the estimated genome size of 45.67 Mb (see above). Manually adjusting the genome may be the most effective way to eliminate contaminants such as bacterial symbionts (including Pseudomonas and Vibrio) and fish hosts in the current assembly (Coyne et al., 2011). After eliminating the redundancy, we obtained a final genome assembly of 45.61 Mb for the *C. irritans*, which is nearly equal to the estimated genome size (Table 1). The draft assembly consisted of 2,384 contigs, and the contig N50 value of our final assembly was 21.24 kb. The summary statistics of *C. irritans* genome assembly compared with other ciliates can be obtained in Supplementary Table S2.

**TABLE 1:** Summary statistics of genome assemblies of *C. irritans*.

Summary statistics of genome assembly	
Total length of genome (Mbp)	45.61
Contig N50 size (Kbp)	21.24
Contig N75 size (Kbp)	14.48
Contig L50 size (Kbp)	689
Contig L50 size (Kbp)	1,379
Contig number (>1,000 bp)	2,384
Contig number (>10000 bp)	2,355
Max contig length (Kbp)	748.71
Gene Prediction and annotation	
Protein-coding gene number	8,729
Mean transcript length (bp)	1,635.71
Mean exons length (bp)	269.56
Mean exons number per gene	6.05

**FIGURE 2:**
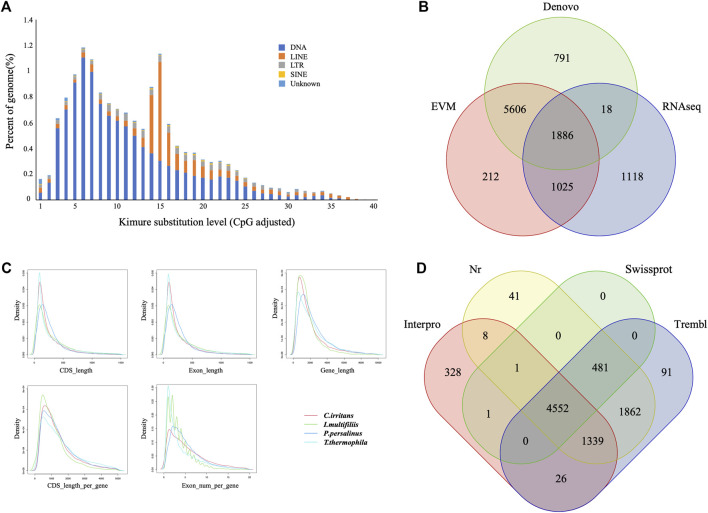
Gene and repetitive element annotations of the *C. irritans* genome. **(A)** Divergence distribution of TEs in the *C. irritans* genome. **(B)** Venn diagram of the number of genes with structure prediction based on different strategies. **(C)** Gene components distribution patterns among *C. irritans* and related species (*I. multifilii*, *P. persalinus*, *T. thermophila*). **(D)** Venn diagram of the number of functionally annotated genes based on different public databases.

Repeat sequences of the *C. irritans* genome were calculated by the combination of both homolog-based and *de novo* methods. There was a total of 4.02 Mb of consensus and nonredundant repetitive sequences obtained by a combination of known, novel and tandem repeats, occupying more than 8.81% of the genome assembly (Supplementary Table S3). DNA transposons accounted for the largest proportion of transposable elements, spanning at least 2.51 Mb accounting for 5.51% of the whole genome. The repetitive sequences are also comprised of long interspersed elements (LINEs; 1.86%), short interspersed nuclear elements (SINEs; 0.10%) and long terminal repeats (LTRs; 0.70%) (Supplementary Table S4). TE accumulation analysis suggested long-term TE actives (Figure 2A). Furthermore, we identified four types of non-coding RNA, 154 miRNAs, 183 tRNAs, 96 rRNAs, and 57 snRNAs in the assembled genome (Supplementary Table S5).

The early-stage gene predictions of the protozoan genome have been largely based on sequence homology (Wang et al., 2003). Gene predictions in newly sequenced species based on the availability of predictions from related species have been used for genome annotation of several protozoa (Mushegian et al., 1998). In order to show sufficiently novel features, new algorithms and strategies need to be developed (Ersfeld, 2003). A total of 8,729 non-redundant protein-coding genes were successfully predicted combining *de novo*, homology searching and transcriptome-assisted predictions in this genome (Figure 2B), with an average gene length of 2.21 kb. The statistics of the gene components were compared with closely homologous species, and the result indicated close distribution patterns in mRNA length, CDS length, exon length and exon number (Figure 2C and Supplementary Table S6). Respectively, 5,034, 8,283, 8,351, and 6,254 genes showed positive hits in Swissprot, NR, TrEMBL and Interpro (Figure 2D and Supplementary Table S7). In order to verify the integrity of the *C. irritans* genome assembly and annotation, we downloaded the *C. irritans* transcriptome sequence published by Lokanathan et al. (2010). BWA (version 0.7.17) and Samtools (v. 1.8) were used to eliminate host contamination and calculate the mapping ratio. As a result, a total of 88.52% of the reads were mapped to this genome. Additionally, we tested completeness by attempting the recovery of conserved single-copy genes from *C. irritans* genome by BUSCO (v. 5.0.0). Out of a database containing 171 single-copy protozoan orthologs, ∼71.4% were fully recovered from the assembly. Similarly, we also tested the published *I. multifiliis* genome (Coyne et al., 2011), and about 82.5% was completely recovered from the assembly (Supplementary Table S8). The test of such conserved single-copy genes in protozoa is inconclusive, which might indicate that some genes are not as conserved in ciliates as they are in vertebrates. The percentage might reflect the evolutionary divergence of the ciliate similar to what has been reported for another protozoon (Porcel et al., 2000). It is necessary to develop algorithms and strategies which are more suitable for the evaluation of protozoan genome integrity.

**FIGURE 3:**
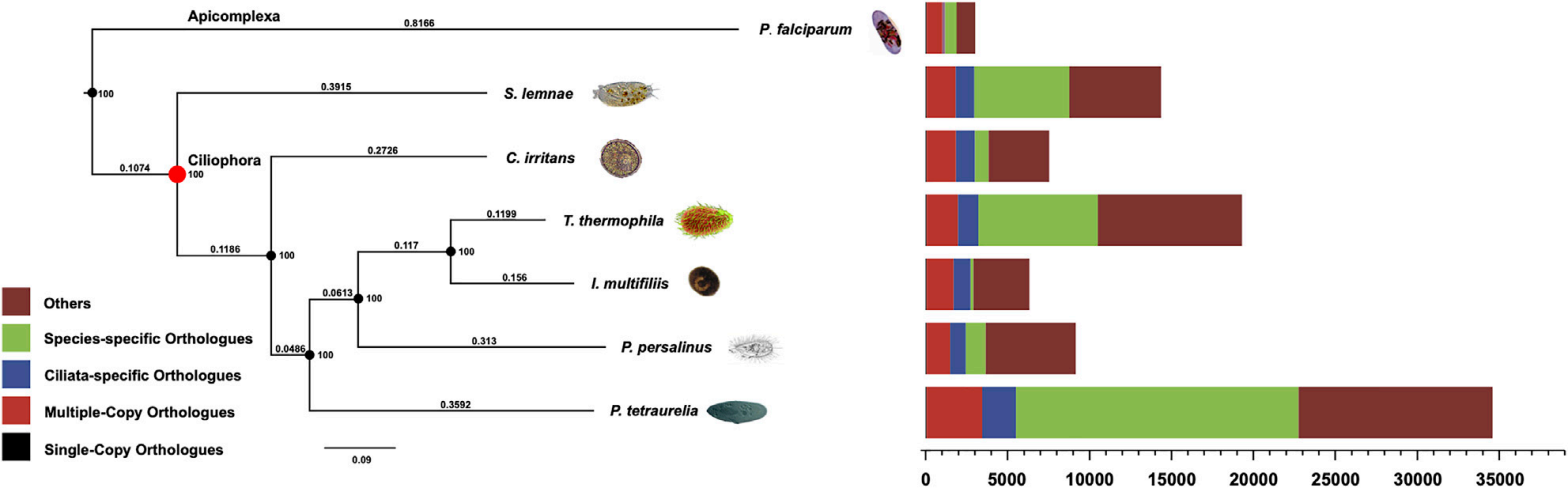
Phylogenetic analysis and distribution of different types of orthologs in representative species (*C. irritans*, *P. falciparum*, *P. persalinus*, *S. lemnae*, *T. thermophila*, *I. multifiliis* and *P. tetraurelia*). The bootstrap value of all branch nodes indicated the robustness of the phylogenetic tree. The numbers above the branches meaning the sequence difference, which were the product of nucleotide mutation rate and divergence time between species.

The systematic position of *C. irritan* has long puzzled taxonomists, and their assignment to the class Oligohymenophorea has been controversial (Lasek-Nesselquist and Johnson, 2019). In order to reveal the phylogenetic relationships, the evolutionary position of *C. irritans* was accessed based on single-copy genes of *C. irritans* and six related species (*P. falciparum*, *P. persalinus*, *S. lemnae, T. thermophila, I. multifiliis* and *P. tetraurelia*). As a result, a total of 15,583 Orthogroups were built and 63 single-copy orthologs in a 1:1:1 manner from all seven protozoa species were used for phylogenetic analysis (Supplementary Table S9). *C. irritans* shared fewer orthologous genes with *P. falciparum* (1,186) than with *I. multifiliis* (2,131) (Supplementary Table S10), which is consistent with its closer morphological resemblance to the latter (Wright and Colorni, 2002). RAxML analyses showed a clear topology in the concatenated tree, that is, with two main evolution nodes are recognizable. *P. falciparum* was used as outgroups, suggesting that it is separated from other species at the class level. The six species, *C. irritans* (Prostomatea), *S. lemnae* (Spirotrichea) and other Oligohymenophorea species which were believed to be members of the phylum Ciliata, were clustered and placed in separate clades (Figure 3). *C. irritans* occupied the basal position within the class, indicating that this species might be an ideal representative to demonstrate the ancestral candidate of the Ciliata (Pan et al., 2019). This is consistent with findings of previous studies based on 18s rRNA sequencing which inferred that *C. irritans* is taxonomically different from *I. multifiliis* (Wright and Colorni, 2002). And the similarity in morphology and development between them may be caused by convergent evolution (Hülsmann and Hausmann, 1994; Zhang et al., 2014).

Inferring phylogenetic relationships based on single genes has certain limitations, and was gradually replaced by other more abundant phylogenetic evidence (Ludwig and Klenk, 2001). With the development of sequencing technology, phylogenetic analysis using whole-genome genetic evidence has been more recognized by researchers (Wu and Eisen, 2008). Similarly, the “Ultra Sequence” was constructed from all the single-copy orthologous genes for phylogenetic tree construction, avoiding many limitations of small data sets (Chen et al., 2019a; Zhou et al., 2019). In our analysis, 63 single-copy orthologous gene sequences of 7 species were used to construct the phylogenomic tree. Therefore, a more accurate estimation of evolutionary relationships could be obtained. However, the current analysis of protozoan genetics is still limited, and expansion of genomic resources is necessary to support future research.

## Conclusion

Here, we used a combination of Illumina and Oxford Nanopore reads to provide the draft genome assembly of *C. irritans.* A total of 8,729 gene structures were annotated using the strategy of multi-evidence combination. The comparative analysis of the gene components distribution showed that *C. irritans* and other closely homologous species have similar distribution patterns. The phylogenetic tree was constructed to illuminate the relationship of the *C. irritans* and six other protozoa. Meanwhile, we demonstrate that Oxford Nanopore can be a very valuable technology to analyze protozoan genomes. The data generated in this study will contribute to facilitate the enlargement of genomic resources for protozoan pathogens, and provide valuable resources for the study of basic genetics and the pathogenic mechanism of parasites.

## Code Availability

The versions, settings and parameters of the software used in this work are as follows:

Genome survey and assembly:

(1) SOAPec: version 2.01; -k 17 -l 52; (2) GenomeScope: version 2.0; all parameters were set as default; (3) NanoFilt: version 2.8.0; -q 9 -l 12000 --headcrop 50 --tailcrop 50; (4) NanoPlot: version 1.33.0; --maxlength 40000 --loglength --plots hex dot pauvre kde; (5) Flye: version 2.9; all parameters were set as default; (6) Racon: version 1.4.3; all parameters were set as default; (7) NextPolish: version 1.4.0; job_type = local; task = 1212; rewrite = no; rerun = 3; sgs_options = -max_depth 100 -bwa; (8) Purge_Dups: version 1.2.5; all parameters were set as default.

Genome annotation:

(1) RepeatMasker: version 4.1.0; -no_is -nolow -norna -gff -poly -html; (2) RepeatModeler: version 2.0.1; -database genome -engine ncbi; (3) TEclass: version 2.1.3; all parameters were set as default; (4) TRF: version 4.09; 2 7 7 80 10 50 500 -m -f -d; (5) Augustus: version 3.4.0; --uniqueGeneId=true --noInFrameStop=true --gff3=on --strand=both; (6) exonerate: version 2.4.0; --model protein2genome --querytype protein --targettype dna --showvulgar no --softmaskquery yes --softmasktarget yes --minintron 20 --maxintron 10000 --showalignment no --showtargetgff yes --showcigar no --geneseed 250 --score 250 --bestn 0 --verbose 0 --geneticcode 6; (7) Transdecoder: version 5.5.0; -t transcripts.fasta -G Tetrahymena; (8) PASA: version 2.4.1; -c alignAssembly.config -C -R -g genome -t transcripts.fasta.clean -T -u transcripts.fasta --ALIGNERS blat,gmap --GENETIC_CODE Tetrahymena; (9) Diamond: version 2.0.6; --query-gencode 6 --outfmt 5; (10) EVidenceModeler: version 1.1.1; --gene_predictions --protein_alignments --transcript_alignments --segmentSize 100000 --overlapSize 10000 –weights weights.txt --stop_codons TGA.

Phylogenetic analysis:

(1) OrthoMCL: version 6.6; all parameters were set as default; (2) MUSCLE: version 3.8.31; parameters: all parameters were set as default; (3) RAxML: version: 8.2.12; parameters: -n sp -m PROTGAMMAAUTO -T 20 -f a.

## Data Availability

The datasets presented in this study can be found in online repositories. The names of the repository/repositories and accession number(s) can be found below: https://figshare.com/, https://doi.org/10.6084/m9.figshare.16922665.v2; https://www.ncbi.nlm.nih.gov/, SRX12890364; https://www.ncbi.nlm.nih.gov/, SRX12890363.

## Abbreviations

BUSCO, benchmarking universal single-copy orthologues; C. irritans, Cryptocaryon irritans; EST, expressed sequence tag; I. multifiliis, Ichthyophthirius multifiliis; L. crocea, Larimichthys crocea; ONT, oxford nanopore technologies; P. falciparum, Plasmodium falciparum; P. persalinus, Pseudocohnilembus persalinus; P. tetraurelia, Paramecium tetraurelia; RNA-seq, RNA sequencing; S. lemnae, Stylonychia lemnae; T. thermophila, Tetrahymena thermophila; Tris-EDTA, Tris–ethylenediaminetetraacetic acid.
