# Supplementary material for: The Draft Genome of Cryptocaryon irritans Provides Preliminary Insights on the Phylogeny of Ciliates
Source: Front Genet. 2022 Jan 12;12:808366. doi: 10.3389/fgene.2021.808366 (PMC8790277; doi:10.3389/fgene.2021.808366)
Supplement: Supplementary file 1 [file Table1.DOCX]

**Table S1.** The data statistic of 17-mer analysis and heterozygosity of *C. irritans* genome.

| Sample | Kmer | kmer  depth | kmer number | GC level (%) | Genome size(M) | Revised Genome size (M) | Heterozygosity (%) | Repeat rate (%) |
| --- | --- | --- | --- | --- | --- | --- | --- | --- |
| tomont | 17 | 92.6061 | 4436461437 | 26.86 | 52.22 | 45.67 | 0.48-0.54 | 8.76 |
