# Supplementary material for: The Draft Genome of Cryptocaryon irritans Provides Preliminary Insights on the Phylogeny of Ciliates
Source: Front Genet. 2022 Jan 12;12:808366. doi: 10.3389/fgene.2021.808366 (PMC8790277; doi:10.3389/fgene.2021.808366)
Supplement: Supplementary file 2 [file Table8.DOCX]

| **Table S8.** Assessment of coding regions completeness by BUSCO. | | | |  |
| --- | --- | --- | --- | --- |
| Special | *C. irritans* | | *I. multifiliis* | |
| Type | Number | Percentage (%) | Number | Percentage (%) |
| Complete BUSCOs (C) | 122 | 71.4 | 141 | 82.5 |
| Complete and single-copy BUSCOs (S) | 95 | 55.6 | 132 | 77.2 |
| Complete and duplicated BUSCOs (D) | 27 | 15.8 | 9 | 5.3 |
| Fragmented BUSCOs (F) | 10 | 5.8 | 9 | 5.3 |
| Missing BUSCOs (M) | 39 | 22.8 | 21 | 12.2 |
| Total BUSCO groups searched | 171 | 100 | 171 | 100 |
