# Supplementary material for: The Draft Genome of Cryptocaryon irritans Provides Preliminary Insights on the Phylogeny of Ciliates
Source: Front Genet. 2022 Jan 12;12:808366. doi: 10.3389/fgene.2021.808366 (PMC8790277; doi:10.3389/fgene.2021.808366)
Supplement: Supplementary file 4 [file Table7.DOCX]

**Table S7.** Functional annotation of the protein-coding genes in *C. irritans* genome.

| **Values** | **Total** | **Swissprot** | **NR** | **TrEMBL** | **Interpro** |
| --- | --- | --- | --- | --- | --- |
| **Number** | 8729 | 5034 | 8283 | 8351 | 6254 |
| **Percentage** | 100% | 57.67% | 94.89% | 95.67% | 71.64% |
