# Supplementary material for: The Draft Genome of Cryptocaryon irritans Provides Preliminary Insights on the Phylogeny of Ciliates
Source: Front Genet. 2022 Jan 12;12:808366. doi: 10.3389/fgene.2021.808366 (PMC8790277; doi:10.3389/fgene.2021.808366)
Supplement: Supplementary file 5 [file Table2.DOCX]

| **Table S2.** Summary statistics of genome assembly between *C. irritans* and other ciliate species. | | | | | | | |
| --- | --- | --- | --- | --- | --- | --- | --- |
| Special | Total length | Largest contig | Contig  number | N50 | L50 | GC (%) | N's per 100 kbp |
| *C. irritans* | 47827192 | 748714 | 2384 | 21237 | 689 | 25.16 | 0.04 |
| *I. multifiliis* | 48799969 | 373042 | 2017 | 64045 | 227 | 15.92 | 174.22 |
| *P. persalinus* | 55455357 | 1988609 | 288 | 367841 | 45 | 18.81 | 47.75 |
| *P. tetraurelia* | 72094543 | 981684 | 697 | 413286 | 64 | 28.05 | 799.45 |
| *T. thermophila* | 103014375 | 2216158 | 1158 | 520943 | 59 | 22.32 | 61.83 |
