# Supplementary material for: The Draft Genome of Cryptocaryon irritans Provides Preliminary Insights on the Phylogeny of Ciliates
Source: Front Genet. 2022 Jan 12;12:808366. doi: 10.3389/fgene.2021.808366 (PMC8790277; doi:10.3389/fgene.2021.808366)
Supplement: Supplementary file 6 [file Table5.DOCX]

| **Table S5.** Summary statistics of non-coding RNA in *C. irritans* genome. | | | | | |
| --- | --- | --- | --- | --- | --- |
| **Type** |  | **Copy** | **Average lengthes (bp)** | **Total lengthes (bp)** | **% of genome** |
| **miRNA** |  | 154 | 107.66 | 165,579 | 0.03466 |
| **tRNA** |  | 183 | 74.81 | 13,691 | 0.02862 |
| **rRNA** | **rRNA** | 96 | 355.115 | 34,091 | 0.07127 |
|  | **5S** | 1 | 63.00 | 63 | 0.00013 |
|  | **18S** | 39 | 403.36 | 157,731 | 0.03289 |
|  | **28S** | 56 | 326.73 | 18,297 | 0.03825 |
| **snRNA** | **snRNA** | 57 | 167.77 | 9,563 | 0.01999 |
|  | **CD-box** | 32 | 151.21 | 4,839 | 0.01011 |
|  | **HACA-box** | 3 | 231.00 | 693 | 0.00144 |
|  | **splicing** | 12 | 166.917 | 2,003 | 0.00418 |
