# Supplementary material for: The Draft Genome of Cryptocaryon irritans Provides Preliminary Insights on the Phylogeny of Ciliates
Source: Front Genet. 2022 Jan 12;12:808366. doi: 10.3389/fgene.2021.808366 (PMC8790277; doi:10.3389/fgene.2021.808366)
Supplement: Supplementary file 7 [file Table3.DOCX]

**Table S3.** Prediction of repetitive elements in the *C. irritans* genome.

| Type | Repeat Size (bp) | % of genome |
| --- | --- | --- |
| Trf | 3,348,345 | 7.00 |
| Repeatmasker | 4,213,938 | 8.81 |
| De novo | 2,773,166 | 5.80 |
