# Supplementary material for: The Draft Genome of Cryptocaryon irritans Provides Preliminary Insights on the Phylogeny of Ciliates
Source: Front Genet. 2022 Jan 12;12:808366. doi: 10.3389/fgene.2021.808366 (PMC8790277; doi:10.3389/fgene.2021.808366)
Supplement: Supplementary file 8 [file Table4.DOCX]

**Table S4. Classification of repeat elements in the *C. irritans* genome.**

| **Type** | ***Denovo*** | | ***Repbase*** | |  | ***Total*** | |  |
| --- | --- | --- | --- | --- | --- | --- | --- | --- |
|  | **Length (Bp)** | **Propration in genome (%)** | **Length (Bp)** | **Propration in genome (%)** |  | **Length (Bp)** | **Propration in genome (%)** |  |
| **DNA** | 2,289,377 | 4.79 | 2,330,995 | 4.87 |  | 2,634,342 | 5.51 |  |
| **LINE** | 175,336 | 0.37 | 199,966 | 0.42 |  | 891,126 | 1.86 |  |
| **LTR** | 109,450 | 0.23 | 134,581 | 0.28 |  | 333,178 | 0.70 |  |
| **SINE** | 36,672 | 0.08 | 3,852 | 0.01 |  | 47,506 | 0.10 |  |
| **Total** | 2,610,835 | 5.47 | 2,669,394 | 5.58 |  | 3,906,152 | 8.17 |  |
