# Supplementary material for: The Draft Genome of Cryptocaryon irritans Provides Preliminary Insights on the Phylogeny of Ciliates
Source: Front Genet. 2022 Jan 12;12:808366. doi: 10.3389/fgene.2021.808366 (PMC8790277; doi:10.3389/fgene.2021.808366)
Supplement: Supplementary file 9 [file Table6.DOCX]

| **Table S6.** Comparison of gene prediction among *C.irritans* and other ciliate species. | | | | | | |
| --- | --- | --- | --- | --- | --- | --- |
| **Species** | **Gene length (bp)** | **Gene number** | **Exon length (bp)** | **Exons number per gene** | **CDS length (bp)** | **CDS length per gene (bp)** |
| ***Cryptocaryon irritans*** | 2210.32 | 8729 | 269.56 | 6.05 | 269.56 | 1629.67 |
| ***Ichthyophthirius multifiliis*** | 1695.16 | 8209 | 359.56 | 3.61 | 359.56 | 1218.78 |
| ***Pseudocohnilembus persalinus*** | 2584.56 | 13179 | 298.02 | 5.53 | 298.02 | 1649.1 |
| ***Tetrahymena thermophila*** | 2399.77 | 26996 | 424.26 | 4.49 | 424.26 | 1878.99 |
